# The involvement of the nuclear lamina in human and rodent spermiogenesis: a systematic review

**DOI:** 10.1186/s12610-018-0072-4

**Published:** 2018-06-20

**Authors:** Marine Paci, Razan Elkhatib, Guy Longepied, Patrice Bourgeois, Pierre F. Ray, Nicolas Levy, Michael J. Mitchell, Catherine Metzler-Guillemain

**Affiliations:** 1 Aix Marseille Univ, Inserm, MMG, U1251, Marseille Medical Genetics, 13385 Marseille, France; 2 APHM Hôpital La Conception, Pôle femmes-Parents-enfants, Centre Clinico-Biologique d’Assistance Médicale à la Procréation-CECOS, 13385 Marseille Cedex 5, France; 3 Genetic Epigenetic and Therapies of Infertility, Institute for Advanced Biosciences, Inserm U1209, CNRS UMR 5309, Université Grenoble Alpes, CHU Grenoble Alpes, F-38000 Grenoble, France

**Keywords:** Nuclear lamina, Lamin, Chromatin, Human, Spermiogenesis, Remodelling, Lamina nucléaire, Lamine, Chromatine, Humain, Spermiogenèse, Remodelage

## Abstract

The nuclear lamina (NL) is a filamentous protein meshwork, composed essentially of lamins, situated between the inner nuclear membrane and the chromatin. The NL is a component of the nuclear envelope, interacts with a wide range of proteins and is required for normal nuclear structure and physiological development. During spermiogenesis the spermatid nucleus is elongated, and dramatically reduced in size with protamines replacing histones to produce a highly compacted chromatin. There is mounting evidence from studies in human and rodent, that the NL plays an important role in mammalian spermatid differentiation during spermiogenesis. In this review, we summarize and discuss the data available in the literature regarding the involvement of lamins and their direct or indirect partners in normal and abnormal human spermiogenesis.

## Résumé

La lamina nucléaire (LN) est un réseau de filaments protéiques, composé essentiellement de lamines, situé entre la membrane nucléaire interne et la chromatine. La LN est un composant de l’enveloppe nucléaire, interagit avec une large gamme de protéines et est nécessaire à l’intégrité de la structure nucléaire et au développement physiologique. Au cours de la spermiogenèse, le noyau de la spermatide s’allonge et sa taille est considérablement réduite, les protamines remplaçant les histones dans le but de constituer une chromatine fortement compactée. De nombreux travaux chez l’homme et chez les rongeurs montrent que la LN joue un rôle important dans la différenciation des spermatides chez les mammifères au cours de la spermiogenèse. Dans cette revue, nous résumons et discutons les données disponibles dans la littérature concernant l’implication des lamines et de leurs partenaires directs ou indirects dans la spermiogenèse humaine normale et anormale.

## Background

Spermiogenesis is the final phase of spermatogenesis, a complex process leading to the formation of haploid spermatozoa from diploid spermatogonia. Spermiogenesis is defined as the differentiation of post-meiotic haploid round spermatids into spermatozoa. During spermiogenesis, the spermatid nucleus is subjected to a unique remodelling of its chromatin involving an extreme compaction of the genome that accompanies a large reduction in nuclear volume with a streamlining of its form from round to elongated and finally pyriform in humans. This remodelling represents a physiological model of nuclear plasticity, orchestrated by dynamic interactions between the nuclear envelope (NE) and the manchette, a network of cytoplasmic microtubules surrounding the nucleus [1]. This plasticity, which is not found in any other cell type, is obviously related to specific properties of the spermatid nucleus and nuclear envelope. Among the NE components, the nuclear lamina (NL) is a meshwork of intermediate filament proteins situated within the nucleoplasm between the chromatin and the inner nuclear membrane. It is a key structure for cellular function and is particularly involved in organising nuclear structure. Over the past 20 years, evidence has emerged supporting the involvement of the NL, and some of its protein partners, in the specific remodelling of the mammalian spermatid nucleus. In this review, after a brief reminder of the nature of the NL and its roles in human somatic pathology, we present the scientific evidence for its involvement during spermiogenesis, initially in mice and then in humans. Finally, we give examples of abnormal human sperm phenotypes that reinforce the importance of the NL and its associated proteins in the control of human physiological spermiogenesis. All articles cited are in English, and were selected using “nuclear lamina, lamin, laminopathies, spermatogenesis and spermiogenesis” as keywords.

## The nuclear lamina

In most cells, the NL is composed of A-type and B-type lamins. The NL meshwork is located at the nuclear periphery through tight interactions of the lamins with a wide range of transmembrane proteins and by a direct association with the membrane of a hydrophobic farnesyl group present at the C-terminus of the B-type lamins [2]. In mammals, three major A-type lamins have been described, A, C and a male meiosis-specific isoform C2. There are also three major B-type lamins, B1, B2 and a spermatid-specific isoform B3. Lamins A and C are expressed in most differentiated cells, and are translated from alternatively spliced transcripts of the lamin A/C gene (LMNA) while lamins B1 and B2 are expressed in nearly all cells and are encoded by distinct genes, the lamin B1 (LMNB1) and lamin B2 (LMNB2) genes respectively [3, 4]. Until recently, lamin isoforms C2 and B3 had only been described in rodent spermatogenesis, where they are expressed through the use of alternative promoters and lack the N-terminal domains of lamin C and B2 respectively [5–7].

The filamentous networks formed by lamins are required for normal nuclear structure and physiological development [8]. Moreover in humans, anomalies of the NL have been identified as the cause of several diseases. Mutations in LMNA encoding A-type lamins are known to underlie the pathogenesis in at least 12 genetic disorders [4] including type 2B1 Charcot-Marie-Tooth disease [9], Hutchinson-Gilford progeria syndrome [10, 11] and mandibuloacral dysplasia [12], dilated cardiomyopathy [13]. Duplications of LMNB1 have been identified in adult-onset autosomal dominant leukodystrophy [14], while mutations in LMNB2 have been associated with acquired partial lipodystrophy [15]. The lamins are known to play varying roles in chromatin organization, nuclear positioning, cell survival, and regulation of DNA replication and transcription in different cell types [8, 16]. These functions are enabled by interactions with protein networks such as the Linker of Nucleoskeleton and Cytoskeleton (LINC) complexes involving Sad1-UNC84 homology (SUN)-domain and Nesprin proteins that can connect the NL to the cytoskeleton and the centrosome, as well as Lamina-associated polypeptide 2, Emerin, Man (LEM)-domain/Barrier-to-Autointegration Factor (BAF) or Lamin B receptor (LBR)/Chromobox (CBX), protein complexes known to connect the NL to the chromatin [17].

### The nuclear lamina is an actor in normal spermiogenesis

The importance of the NE in the remodelling that occurs during spermiogenesis was first suggested by the abnormal development of the sperm head, acrosome and flagellum in mutant mouse lines whose spermatids lack the expression of either Lis1 or Mgcl-1, proteins that, respectively, connect the NL to the cytoskeleton, and the NL to the chromatin: Lis1 regulates the interaction of the LINC complex and the dynein motor, while Mgcl-1 is a component of the NE and a binding partner of the lamina-associated polypeptide 2, isoform beta, LAP2β [18, 19]. Recently a more direct link between human infertility and the nuclear envelope has been established through the study of *DPY19L2*, a gene transcribed predominantly in spermatids. In human, both copies of the *DPY19L2* gene are deleted in around 70% of men with globozoospermia, a rare phenotype characterised by malformed round sperm heads without an acrosome [20–22]. In the mouse, the knockout of DPY19L2 produced an identical phenotype, and the DPY19L2 protein was shown to localise to the region of the inner nuclear membrane facing the acrosome. Interestingly, the NL is excluded from this region, inferring an essential interaction between the acrosome, DPY19L2 and the NL [23]. Furthermore, the sperm nucleus from *Dpy19l2* KO mice was shown to be poorly compacted with a failure to replace the histones with the protamins [24]. These findings indicate that the NL may be involved in diverse aspects of spermatid differentiation during spermiogenesis, from the remodelling of nuclear morphology and the chromatin to the formation and positioning of the acrosome and the flagellum.

**Fig. 1:**
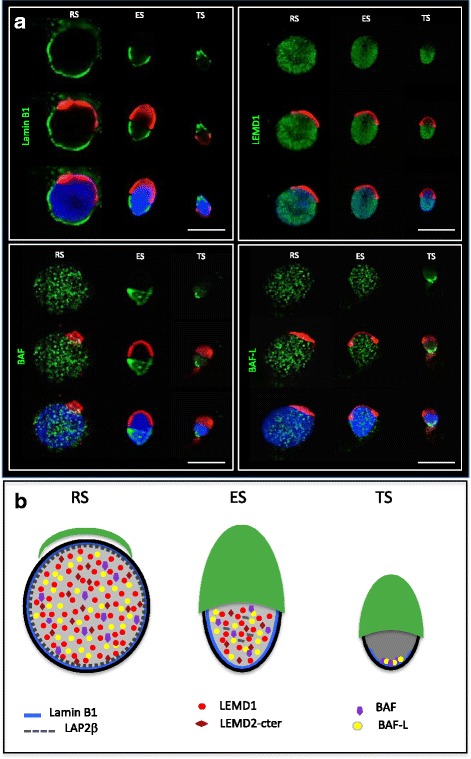
**a** Immunolocalisation of Lamin B1, LEMD1, BAF and BAF-L (green) on human spermatids and testicular spermatozoa. Labelling is shown on successive steps of spermiogenesis: round spermatid (RS), elongating spermatid (ES), testicular spermatozoa (TS). The acrosome is identified using lectin PNA (red) and DNA is counterstained with DAPI (blue). Scale bar is 10 μm. **b** Schematic representation of nuclear lamina proteins and nuclear partner proteins during human spermiogenesis. Successive steps are represented: round spermatid (RS), elongating spermatid (ES), testicular spermatozoa (TS). The acrosome is represented in green. LEMD1: LEM domain containing 1; LEMD2-Cter: LEM domain containing 2-Cterminal region; BAF: Barrier-to-Autointegration Factor; BAF-L: Barrier-to-Autointegration Factor Like; LAP2b: LAP2: lamina-associated polypeptide 2-β isoform

The first studies to characterize the NL during spermiogenesis focused on rodents. They showed that the A-type lamins, A and C, are absent from rodent spermatids, and that the NL is composed exclusively of B-type lamins [25, 26]. Lamin B1 and the B3 spermiogenesis specific isoforms were localised at the nuclear periphery in spermatids, but lamin B2 was not detected [27]. During rodent spermiogenesis, the distribution of lamin B1 and B3 changes, with a progressive regression to the posterior pole of the elongating spermatid nucleus as the acrosome spreads, and neither is present in mature spermatozoa [27, 28]. It has also been shown that the overexpression of mouse lamin B3 causes severe nuclear deformation in cultured cell lines [5, 29]. Based on this finding it has been hypothesised that the role of lamin B3 in spermatids is to increase the flexibility of the NL to enable the intense nuclear remodelling that occurs during spermiogenesis [29]. In humans, the first characterization of the NL structure during human spermiogenesis was reported in 2015 by our team, describing the expression pattern and localization of A- and B-type lamins in post-meiotic human male germ cells [30], (Fig. 1a and b). We identified a transcript encoding a B3 isoform expressed in human spermatids and showed that the lamins B1 and B2/B3 are the only lamins present during human spermiogenesis. Definitive proof that the lamin B3 protein is produced in human spermatids requires the production of a specific antibody.

We further showed that, as in the mouse, the ectopic expression of human lamin B3, but not lamin B2, induces a major nuclear shape deformation in HeLa cells. In human spermatids, B-type lamins are present at the nuclear periphery, except in the region covered by the acrosome. As the spermatid matures, the B-type lamin label recedes towards the flagellum, remaining detectable on around 40% of mature ejaculated spermatozoa. Our results in human demonstrate the similarities in the composition and behaviour of the spermatid NL between rodents and humans and provide strong evidence that the shared features are of functional significance during mammalian spermiogenesis. However, the precise role of the lamin B3 isoform during spermiogenesis remains to be determined.

### Lamin partners are involved in human spermiogenesis

There is now considerable evidence for the existence of functional links between the NL and the cytoskeleton through SUN proteins. In contrast it is emerging that the links characterised in somatic cells between the NL and the chromatin compartment, via LEM-domain protein/BAF complexes or LBR/CBX complexes, may be absent after the earliest stage of spermiogenesis.

The SUN-domain and the KASH-domain protein families form protein complexes that connect the nucleus to the major cytoskeleton network [31, 32]. In spermatids, the proteins SUN3, SUN4 and SUN5 have a localisation at the nuclear periphery coincident with the lamins [33–36]. In mice lacking SUN4, the acrosome remains attached and the chromatin condenses, but the manchette does not attach to the nucleus, spermatids do not elongate and mature spermatozoa have round heads [35, 37].

A number of proteins are known to form links between the NL and the chromatin in somatic cells: Lamin B Receptor (LBR), members of the LEM-domain (the Lamina-associated polypeptide 2, Emerin, MAN1 domain) family and Barrier-to-Autointegration Factor (BAF) [38–40]. LBR and most LEM-domain proteins are inner nuclear membrane proteins that through their interaction with the nuclear lamina participate in chromatin organization, nuclear pore complex assembly, nuclear positioning, nuclear structure, nuclear envelope breakdown and reassembly during mitosis, DNA replication, transcriptional regulation and signal transduction. The Barrier-to-Autointegration factor (BAF) is a conserved chromatin protein capable of simultaneously binding both DNA and the LEM-domain of some LEM-domain proteins [41]. LBR (Lamin B Receptor) interacts with CBX heterochromatin proteins, DNA and free histones [42, 43]. In the rat, LBR has been localised to the nuclear periphery of elongating spermatids, and it has been suggested that LBR could be involved in chromatin remodelling during spermiogenesis, based on the demonstration that in vitro it interacts with Protamine 1 [44]. Human LEM-domain proteins are a heterogeneous family of mainly nuclear proteins that share a conserved amino acid domain, the LEM-domain, that is a binding site for BAF (barrier-to-autointegration factor). In addition to the founding proteins, four further human LEM-domain encoding genes have been described: LEM domain containing 1 (LEMD1) expressed predominantly in the testis [45], LEM domain containing 2 (LEMD2) [46], Ankyrin repeat and LEM domain containing 2 (ANKLE2) [47] and Ankyrin repeat and LEM domain containing 1 (ANKLE1). BAF has been shown to be able to interact simultaneously with DNA and the LEM-domain of Lamina-associated polypeptid 2 (LAP2) and Emerin genes [48, 49], indicating that BAF-LEM complexes serve to link the chromatin to the nuclear periphery. It has been shown that LAP2 isoforms are present in rat spermatids with LAP2β predominating during spermiogenesis, but with only LAP2α being retained in mature spermatozoa [28]. ANKLE1 is an endonuclease, ANKLE2 (also known as LEM4) is the only LEM-domain protein that does not localise to the interphase nucleus, and it is found at the cytoplasmic face of the endoplasmic reticulum, where it controls post-mitotic formation of the nuclear envelope by regulating BAF phosphorylation [50]. BAF is expressed widely, but has a paralogue, barrier-to-autointegration factor-like (BAF-L) that is expressed predominately in testis and pancreas [51, 52].

During human spermiogenesis, we characterised the known lamina-chromatin interface proteins and showed that transcripts for Emerin, LEMD1, LEMD2, ANKLE2, LAP2α, LAP2β, BAF-L and LEMD2 (only the 3′ end of the coding region) were detected in human spermatozoa. However, transcripts for LBR, LEMD3 and ANKLE1 were not detected, and consistent with their absence during human spermiogenesis we tested for, but did not detect LBR or LEMD3 proteins in spermatids by immunofluorescence [53]. At the protein level in spermatids, no protein localised to the nuclear periphery, LEMD1, LEMD2-Cter, LAP2β, BAF and BAF-L were detected in the nucleoplasm, receding towards the posterior pole as spermatids mature (Fig. 1a and b), whereas ANKLE2 was detected in the cytoplasm, localising to the endoplasmic reticulum in round spermatids. These data establish that the lamina-chromatin interface in human spermatids is radically distinct from that defined in somatic cells. Recently, the lamina associated polypeptide 1 (LAP1) was reported to be located at the centriolar pole of elongated spermatids [54]. In ejaculated spermatozoa, only BAF and BAF-L can be detected, suggesting that they might contribute to the shaping of the spermatozoon nucleus, and perhaps, after fertilization, to male pronucleus formation.

## The NL in abnormal human spermiogenesis

As previously described, homozygous deletions of DPY19L2, a gene encoding an inner nuclear membrane protein, are frequently found in cases of globozoospermia, explaining 25–70% of cases [22]. In the mouse, knockout of Dpy19l2 alters the organisation of the NL, as evidenced by the persistence of lamin B1 throughout the nuclear periphery in round spermatids, the failure of spermatozoa nuclear shaping and the detachment of the acrosomal vesicle, a phenotype identical to that found in men deleted for *DPY19L2* [23, 55, 56]. Even though nothing is known about how DPY19L2 and the NL interact, it has been proposed that DPY19L2 may function as a LINC-like protein during mammalian spermiogenesis [57]. It has been reported that the NL also appears immature in human globozoospermic spermatozoa, with a lamin B1 signal predominantly observed at the whole nuclear periphery, not polarized as in control spermatozoa [58]. Thus DPY19L2 may play a role in displacing the lamina from the nuclear periphery under the acrosome in spermatids. Moreover, BAF and BAF-L are not detected in globozoospermic spermatozoa. So, the lack of maturation of the NL, and the modifications in the expression or location of chromatin-partners might underlie the sperm chromatin defects and the chromatin heterogeneity observed in globozoospermia.

Decapitated spermatozoa represent another rare form of teratozoopermia [59, 60]. In a recent study, 8 of 17 men with this phenotype were found to carry a rare potentially damaging variant on each allele of the SUN5 gene, providing strong evidence that the loss of SUN5 function causes acephalic spermatozoa syndrome [61]. We strengthened this conclusion with the report of a homozygous deletion of SUN5 in three related men, the second case of a biallelic high confidence loss-of-function mutation, confirming that a loss of SUN5 function is the cause of acephalic spermatozoa [62]. These data show that SUN5 is required for the formation of the sperm head-tail junction and male fertility. Interestingly SUN5 co-localises with the NL throughout spermatid differentiation and, in the mature spermatozoa, is detected at the posterior pole of the sperm head where the flagellum is joined to the nucleus.

## Conclusion

A considerable amount of data from human and rodents highlights the importance of the NL during mammalian spermiogenesis. As in somatic cells, the NL must be considered as an essential determinant in the management of germ cell differentiation during spermiogenesis, and thus as critical for the production of spermatozoa competent to fertilize and induce development of a viable embryo and a healthy individual. Components of the NL or some of its direct and indirect partners may represent new positive biomarkers of human spermatozoa quality. Further experiments are now needed to clarify the degree to which these markers contribute to human spermatozoa quality.

## Abbreviations

ANKLE1: Ankyrin repeat and LEM domain containing 1
ANKLE2: Ankyrin repeat and LEM domain containing 2
BAF: Barrier-to-autointegration factor
BAF-L: Barrier-to-autointegration factor like
CBX: Chromobox
DPY19L2: Dpy-19 like 2
KASH: Klarsicht, ANC-1 and syne homology
LAP1: lamina-associated polypeptide 1
LAP2: lamina-associated polypeptide 2
LBR: Lamin B receptor
LEM: Lamina-associated polypeptide 2, Emerin, Man
LEMD1: LEM domain containing 1
LEMD2: LEM domain containing 2
LINC: Linker of nucleoskeleton and cytoskeleton
LMNA: Lamin A/C
LMNB1: Lamin B1
LMNB2: Lamin B2
LN: Lamina nucléaire
NE: Nuclear envelope
NL: Nuclear lamina
SUN: Sad1-UNC84 homology
